# Endemicity of *Toxoplasma* infection and its associated risk factors in Cebu, Philippines

**DOI:** 10.1371/journal.pone.0217989

**Published:** 2019-06-12

**Authors:** Rochelle Haidee D. Ybañez, Chadinne Girlani R. Busmeon, Alexa Renee G. Viernes, Jorim Z. Langbid, Johanne P. Nuevarez, Adrian P. Ybañez, Yoshifumi Nishikawa

**Affiliations:** 1 National Research Center for Protozoan Diseases, Obihiro University of Agriculture and Veterinary Medicine, Obihiro, Hokkaido, Japan; 2 College of Science, University of the Philippines Cebu, Lahug, Cebu City, Philippines; 3 Institute of Molecular Parasitology and Protozoan Diseases at Main Campus and College of Veterinary Medicine at Barili Campus, Cebu Technological University, Cebu City, Philippines; 4 College of Medicine, University of the Visayas, Banilad, Cebu City, Philippines; Universita degli Studi di Parma, ITALY

## Abstract

*Toxoplasma gondii* is a single-celled intracellular apicomplexan parasite that causes toxoplasmosis. It is capable of infecting humans and nearly all warm-blooded animals including pigs, but cats are the only known definitive host. This ubiquitous zoonotic pathogen can cause abortion, stillbirth and fetal abnormalities, and has been associated with mental and behavioral changes in humans. Acute infection is potentially fatal in immunocompromised individuals. The present study aimed to assess the *Toxoplasma* seroprevalence in pigs, humans and cats after its initial reported detection in pigs about three decades ago in Cebu, Philippines. A total of 924 humans, 104 cats and 514 slaughter pigs were tested for antibodies against *T*. *gondii* using a commercial latex agglutination test. The results revealed positive detection rates of 26.3% (244/924) for humans, 42.3% (44/104) for cats and 13.4% (69/514) for slaughter pigs. Statistical analyses revealed that the area *(P* = 0.004), cat ownership (*P* = 0.020), the frequency of contact with cats (*P* < 0.0001) and consumption of street foods (*P* = 0.043) were significantly associated with seropositivity for *T*. *gondii* in humans. Meanwhile, the use of litter trays (*P* = 0.001) and contact with other animals (*P* = 0.007) were significantly associated with seropositivity in cats. The odds ratio for selected significant factors revealed that living in suburban areas (OR 1.66, 95% CI: 1.20–2.31), owning a cat (OR 1.482, 95% CI: 1.07–2.07) and eating street foods (OR 1.585, 95% CI: 1.01–2.48) were associated with an increased risk of *T*. *gondii* exposure in humans. In cats, the use of a litter tray (OR 4.5, 95% CI: 1.73–11.71) was associated with an increased risk of exposure. None of the profile parameters were found to be significantly associated with seropositivity in slaughter pigs (*P* > 0.05). This study is the first report of the serological detection of *T*. *gondii* in humans and cats in Cebu, Philippines, and the first assessment of the prevalence of the parasite in pigs in the area since its initial detection in 1982. This is also the first report documenting the seropositivity of *T*. *gondii* in pregnant women in the country. The confirmed seropositivity of *T*. *gondii* in Cebu, Philippines, in the present study implies the endemicity of toxoplasmosis in this area and highlights the need for routine testing and increased public awareness.

## Introduction

Toxoplasmosis is a public health problem worldwide. It is caused by *Toxoplasma gondii*, an obligate, intracellular, parasitic protozoan. It is zoonotic and is capable of infecting nearly all warm-blooded animals including humans, but cats are the only known definitive host. *T*. *gondii* reproduces in the gut of felids. After reproduction, the oocysts are shed through the feces. These environmentally-resistant oocysts can then contaminate the soil, which may be ingested by an intermediate host, such as rodents, birds or other warm-blooded animals [1]. This parasite can cause infections in humans following the ingestion of raw meat infected with tissue cysts, food or drink contaminated with oocysts, or by direct assimilation from the environment [2,3]. Vertical transmission from the infected mother to the fetus may also occur [4]. *T*. *gondii* infections in humans are generally asymptomatic or are manifested by flu-like symptoms and lymphadenopathy [5]. In young children, pregnant women, congenitally-infected fetuses and newborns, and immunocompromised people such as those with HIV/AIDS, those undergoing chemotherapy or those who have received an organ transplant, toxoplasmosis may be severe [2,6]. In most livestock animals such as poultry, pigs, and cattle, clinical toxoplasmosis is rare [2]; however, just like in humans, it mostly affects the young and fetuses. Thus, it is considered one of the most common zoonotic infections causing abortions in both humans and female animals [7–8]. Clinical signs rarely present themselves in infected cats, and if present, are caused by tissue inflammation and necrosis induced by the intracellular growth of tachyzoites [9]. Like most human and animal cases, congenital infections in cats are more severe than in adults [9–10].

The course of *T*. *gondii* infection may be affected by different conditions, such as the different sources and degrees of exposure to the parasite, and the immunological status of the individual [11]. Cases also vary between countries and regions, being affected by other factors [12] such as the climate, which affects the conditions for survival of the oocysts in the environment, and anthropogenic factors as reflected by the individual’s hygiene and diet [13]. Other risk factors for acquiring human *T*. *gondii* infection, include age [14–16], sex [17], close association with cats [18–20], cleaning cat litter [21] and a lower educational level [18, 19]. In some areas, seroprevalence has been reported to vary among residents of rural, suburban and urban areas, with significantly higher seroprevalence in rural and suburban areas than in urban areas [15,20]. This variation may be attributed to the difference in socioeconomic status and hygienic practices, factors which are linked to the ingestion of oocysts from contaminated water, crops, animal products, and unpasteurized milk, likely contaminated from oocysts shed by cats or contaminated soil [15,16]. Generally, contact with cats and consumption of raw or undercooked meat were the most common risk factors in acquiring toxoplasmosis.

For non-feline animals, contact with cats increases an animal’s susceptibility to infection, which may be through direct contact or by indirect contact through a water source [22]. In pigs, the farming system affects the *Toxoplasma* seroprevalence [23,24]. Serodetection is significantly higher among extensively farmed pigs compared with intensively farmed pigs [2] as a result of outdoor roaming, which increases contact with infected rodents, dead animals and oocyst-contaminated feed, water and surfaces [24]. For cats, seroprevalence increases with age as a result of increased contact with infection sources, but younger cats can also become infected [25]. Poor body condition [25–26], access to the outdoors, the presence of dogs in the household, hunting, a history of being stray, and feeding on raw meat [27–28] have been identified as risk factors for infection in cats. Studies also reported that location (urban, suburban and rural areas), diet and domestication [29] influence seropositivity in animals. Table food may contain tissue cysts thereby causing infection in cats [27, 29].

The Sabin–Feldman *Toxoplasma* dye test is considered the gold standard for detecting anti-*Toxoplasma* antibodies in humans. As the parasite is not always detectable in patient samples, serological tests such as an enzyme-linked immunosorbent assay (ELISA), a latex agglutination test (LAT) and an immunochromatographic test (ICT) are mostly used. For detection of the organism in patient samples, PCR is widely used [8].

Toxoplasmosis has been reported in many different countries in Southeast Asia. In the Philippines, studies that have documented toxoplasmosis have mainly used serological methods, with several reports in pigs, rats, cats and a few histopathological studies in rats and cats [30–32]. To date, three studies have documented *T*. *gondii* infections in humans in Metro Manila, Mindoro, Cavite, Samar, and Leyte [20,33,34]. In Cebu, the only report was in slaughter pigs in 1982, which documented a detection rate of 29.5% [35]. There are no studies yet on toxoplasmosis in humans and cats in this area. Thus, this study aimed to update the epidemiological status of the seroprevalence of *T*. *gondii* in humans, pigs, and cats in Cebu. It also sought to assess risk factors associated with the infection to increase the awareness of toxoplasmosis as a major public health threat in Cebu, Philippines.

## Materials and methods

### Sample population and sampling area

**Fig 1:**
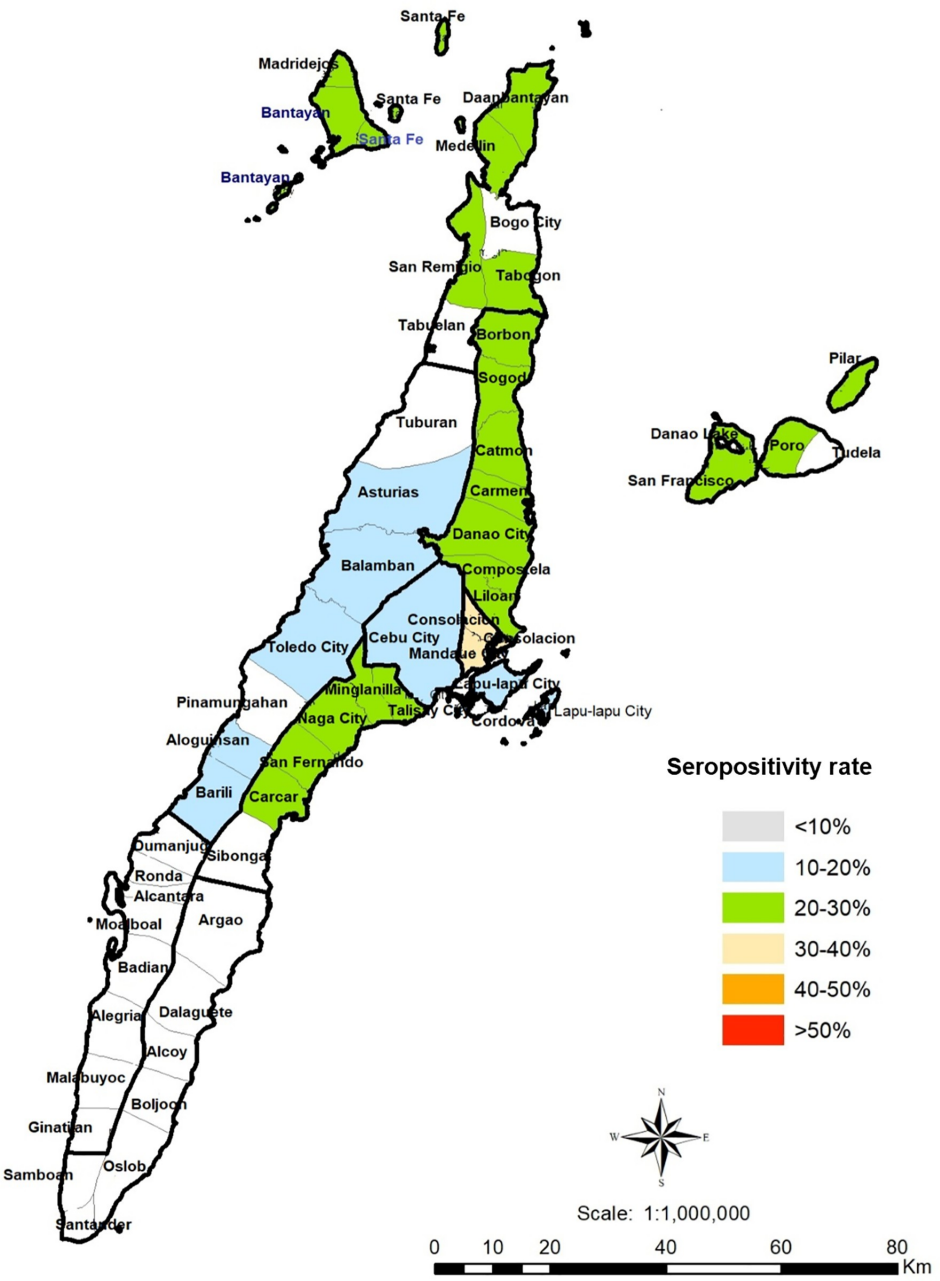
Geographical distribution of *T*. *gondii* infections of humans in Cebu, Philippines. Colored areas are those with *T*. *gondii*-seropositive respondents. Urban: Cebu City, Mandaue City, Lapu-Lapu City; Suburban: Minglanilla, Talisay, Naga, San Fernando, Carcar, Consolacion, Liloan, Danao, Compostela; Rural: Barili, Argao, Alegria, Dumanjug, Daanbantayan, Carmen, Sogod, Catmon, San Remigio, Borbon, Bantayan Island, Bogo, Madridejos, Poro, San Francisco, Danao Lake, Pilar. QGIS software, a free and open source geographic information system, was used to create the map (https://www.qgis.org/). PhilGIS, an open and free source for GIS data for the Philippines, was used to integrate the municipal boundaries or base layer (http://philgis.org/province-page/cebu).

**Fig 2:**
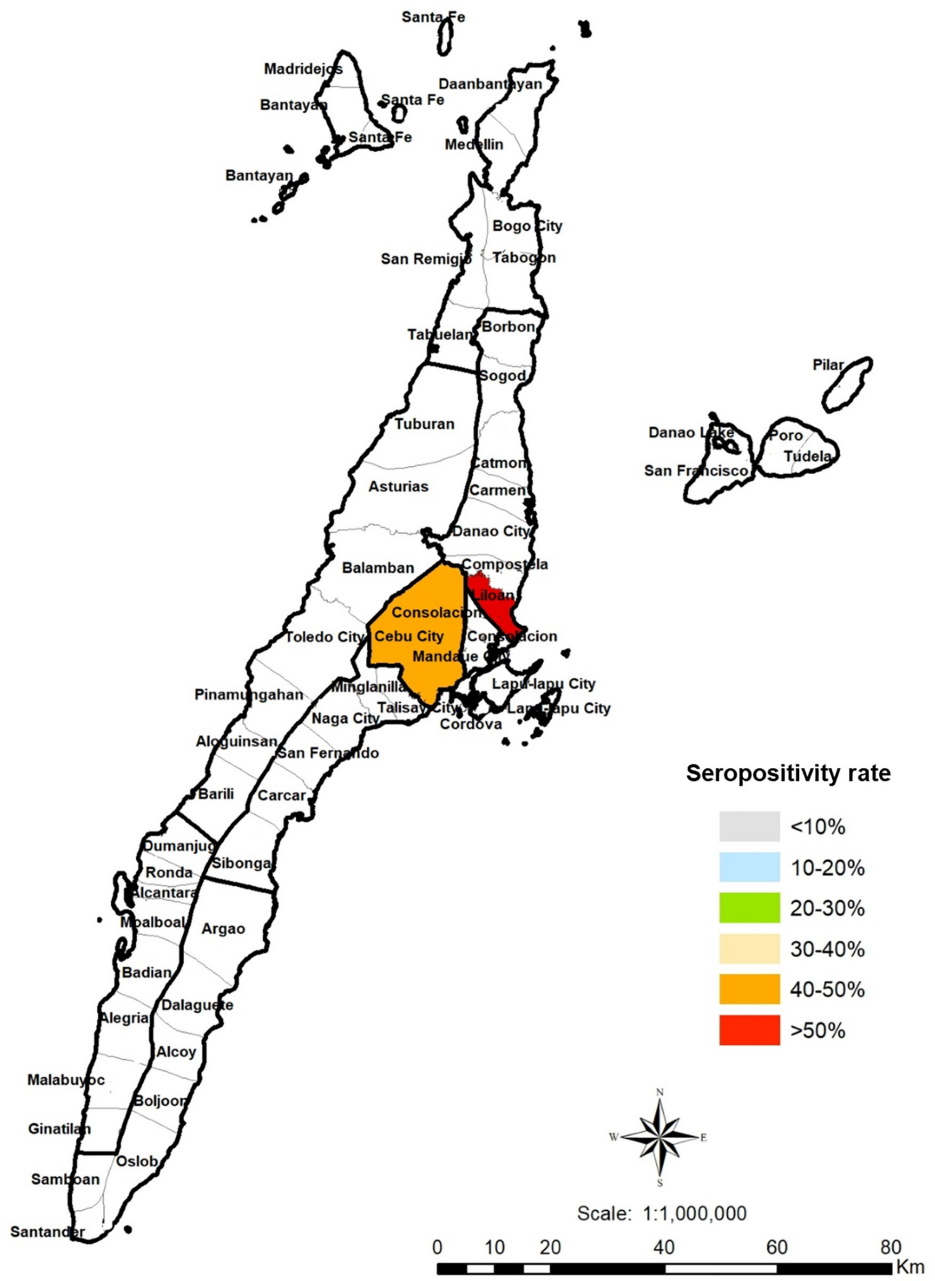
Geographical distribution of *T*. *gondii* infections in cats in Cebu, Philippines. Colored areas are those with *T*. *gondii*-seropositive cats. QGIS software was used to create the map (https://www.qgis.org/). PhilGIS was used to integrate the municipal boundaries or base layer (http://philgis.org/province-page/cebu).

**Fig 3:**
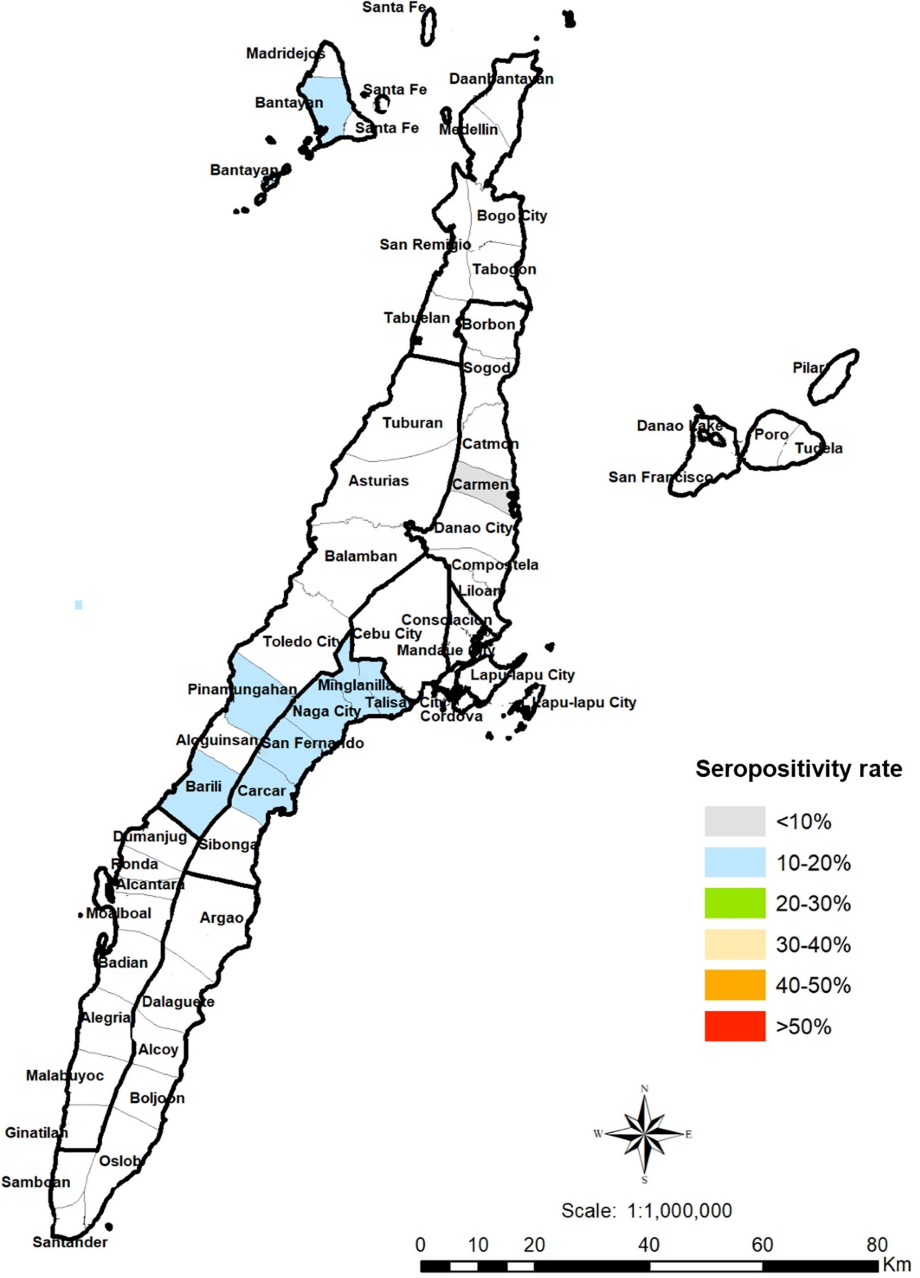
Geographical distribution of *T*. *gondii* infections in slaughter pigs in Cebu, Philippines. Colored areas are those with *T*. *gondii*-seropositive pigs. QGIS software was used to create the map (https://www.qgis.org/). PhilGIS was used to integrate the municipal boundaries or base layer (http://philgis.org/province-page/cebu).

A total of 594 humans, 514 pigs and 104 cats were sampled in Cebu, Philippines. For humans, adult participants (18 years old and above) from rural, suburban and urban areas in Cebu were chosen by convenience sampling (Fig 1). The respondents must have been a resident of the area for at least six months to be included. Respondents were recruited from September 2016 to April 2018. The cat samples came from households, rescue shelters and the streets of Cebu City and Liloan, Cebu, Philippines (Fig 2). The pig samples were obtained from Talisay City Slaughterhouse, Talisay City, Cebu, Philippines. The sampled pigs came from different areas in the province (Talisay, Pinamungajan, Barili, San Fernando, Cebu City, Naga City, Carcar, Bantayan Island, Minglanilla, Carmen and Compostela) (Fig 3). The pigs had slaughter weights of 95–110 kg. Cat and pig serum samples were collected during April 2018. The respondents and animal profiles and risk factors were determined using survey questionnaires (S1–S3 Figs).

### Profile parameters

Out of 924 respondents, most were women (60.9%) who were not pregnant (96.8%), single (63%), students (52.6%), and residing in an urban area (58%). The study population comprised predominantly of the younger age group (mean age = 32 years). This is representative of the general population in the Philippines, which has a large proportion of younger people. The majority of the respondents had no current (88.1%) or previous medical conditions (92.5%) and most consumed street foods (85%). The majority also did not have a pet cat (38.3%) or had no contact with cats (38.3%) (Table 1).

**Table 1:** Seroprevalence of toxoplasmosis in humans in Cebu, Philippines (N = 924).

Profile	Positive (N = 244)	Prevalence %	OR	95% CI	*P*-value
**Sex**					0.611
Female (N = 563)	152	27.0	1.00		
Male (N = 361)	92	25.5	1.08	0.80–1.46	
**Age**					0.472
18–30 years (N = 556)	149	26.8	1.00		
31–40 years (N = 155)	34	21.9	0.77	0.50–1.17	
41–50 years (N = 105)	27	25.7	0.95	0.59–1.52	
51–60 years (N = 52)	17	32.7	1.33	0.72–2.44	
61–70 years (N = 41)	11	26.8	1.00	0.49–2.05	
71 years up (N = 15)	6	40.0	1.82	0.64–5.20	
**Civil Status**					0.971
Single (N = 582)	153	26.3	1.00		
Married (N = 321)	85	26.5	1.01	0.74–1.38	
Widowed (N = 21)	6	28.6	1.12	0.43–2.94	
**Area**					0.004*
Urban (N = 536)	131	24.5	1.00		
Rural (N = 148)	27	18.6	0.70	0.44–1.12	
Sub-urban (N = 240)	86	35.1	1.66	1.20–2.31	
**Occupation**					0.060
Health related (N = 169)	32	18.9	1.00		
Non-health related (N = 231)	56	24.2	1.37	0.84–2.18	
Student (N = 486)	136	28.0	1.66	1.08–2.56	
Preferred not to say (N = 38)	20	52.6	4.76	2.26–10.01	
**Present medical condition**					0.109
No (N = 814)	208	25.6	1.00		
Yes (N = 110)	36	32.7	1.42	0.92–2.18	
**Previous medical condition**					0.829
No (N = 855)	225	26.3	1.00		
Yes (N = 69)	19	27.5	1.06	0.61–1.84	
**Has pet cats**					0.020*
No (N = 354)	95	26.8	1.00		
Yes (N = 301)	106	35.2	1.48	1.07–2.07	
Preferred not to say (N = 269)	43	16.0	0.52	0.35–0.78	
**Frequency of contact with cats**					<0.0001*
None (N = 412)	105	25.5	1.00		
Less frequent (N = 278)	35	12.6	0.42	0.28–0.64	
Frequent (N = 229)	104	45.4	2.43	1.73–3.42	
Prefer not to say (N = 5)	0	0.0	0.27	0.01–4.48	
**Consumption of street foods**					0.043*
No (N = 139)	27	19.4	1.00		
Yes (N = 785)	217	27.6	1.58	1.01–2.48	
**Pregnant Women**					0.316
No (N = 545)	149	27.3	1.00		
Yes (N = 18)	3	16.7	0.53	0.15–1.86	0.323

Odds ratio (OR) was calculated by comparison with the first of each factor.

*Statistically significant with *T*. *gondii* seropositivity (*P*-value < 0.05)

Only one cat out of 104 tested in this study showed poor body condition. The majority of the cats were male (54.8%), in rescue shelters (47.1%), not neutered or spayed (60.6%), and were fed a mixed table food diet (53.8%). Furthermore, the majority of cats had no litter trays (75%), were allowed outdoors (85.6%) and had contact with other animals (95.2%) (Table 2). By comparison, most of the pigs tested were male (55.8%) and were raised in extensive or backyard farms (55.6%) (Table 3).

**Table 2:** Serological survey of toxoplasmosis in cats in Cebu, Philippines (N = 104).

Profile	Positive (N = 44)	Prevalence %	OR	95% CI	*P*-value
**Sex**	** **	** **			
Female (N = 47)	22	46.8	1.00		
Male (N = 57)	22	38.6	1.40	0.64–3.06	0.400
**Type**					
Household or domesticated (N = 47)	21	44.7	1.00		
0Rescued or in shelter (N = 49)	16	32.7	1.67	0.73–3.82	0.230
Stray (N = 8)	7	87.5	0.12	0.01–1.01	0.051
**Neutered/Spayed**					
No (N = 63)	29	46.0	1.00		
Yes (N = 41)	15	36.6	0.68	0.30–1.51	0.340
**Body condition**					
Good (N = 103)	44	42.7	1.00		
Bad (N = 1)	0	0.0	0.45	0.02–11.20	0.623
**Diet**					
Commercial food (N = 25)	13	52.0	1.00		
Table food (N = 23)	9	39.1	1.69	0.53–5.31	0.373
Raw meat (N = 0)	0	0.0			
Mixed (N = 56)	22	39.3	1.67	0.65–4.33	0.288
**Use of litter trays**					
No (N = 78)	26	33.3	1.00		
Yes (N = 26)	18	69.2	4.50	1.73–11.71	0.002*
**Access to outdoors / hunting**					
No (N = 9)	6	66.7	1.00		
Yes (N = 95)	38	40.0	0.33	0.08–1.41	0.136
**Contact with other animals**					
No (N = 4)	4	100.0	1.00		
Yes (N = 100)	40	40.0	0.07	0.00–1.42	0.084

Odds ratio (OR) was calculated by comparison with the first of each factor.

*Statistically significant with *T*. *gondii* seropositivity (*P*-value < 0.05)

**Table 3:** Seroprevalence of toxoplasmosis in slaughter pigs in Cebu, Philippines (N = 514).

Profile	Positive (N = 69)	Prevalence %	OR	95% CI	*P*-value
**Sex**					
Female (N = 227)	32	14.1	1.00		
Male (N = 287)	37	12.9	0.91	0.54–1.50	0.69
**Rearing system**					
Intensive / Concrete (N = 228)	28	12.3	1.00		
Non-intensive / Backyard (N = 286)	41	14.3	1.20	0.71–2.00	0.50

Odds ratio (OR) was calculated by comparison with the first of each factor.

### Blood sampling and serum collection

Blood samples were aseptically collected by venipuncture of the cephalic vein for humans and the jugular vein for cats by a licensed phlebotomist and veterinarian, respectively. Blood from pigs was obtained in the bleeding process during slaughter. Serum samples were separated by centrifugation and stored at ˗20°C until further use. The profile of participants was obtained using a questionnaire that asked for relevant information, including some risk factors for toxoplasmosis.

### Testing of sera using a latex agglutination test (LAT)

The sera were tested for antibodies against *T*. *gondii* using a commercial LAT kit (Toxocheck-MT; Eiken Chemical, Tokyo, Japan). The test was performed according to the manufacturer’s recommendation. Briefly, 25 μL of latex solution was added into a round-bottom 96-well plate and 63 μL of LAT buffer into another round-bottom 96-well plate. The serum sample (1 μL) was added to the LAT buffer. After pipetting the serum and LAT buffer to mix, 25 μL of the serum-buffer mixture (1:64) was added to 25 μL of latex solution. The plate was sealed and allowed to stand at room temperature for 12 h. Agglutination or visible clumping spreading throughout the well was interpreted as a positive reaction. A small, distinct circular precipitation in the center was indicative of a negative response. Positive and negative reference serum samples used in the assay were sera confirmed to be positive or negative using a commercially available ELISA kit (Platelia Toxo IgG/IgM; Bio-Rad, Hercules, CA, USA).

### Data processing and analysis

Data from the questionnaire and LAT results were manually tabulated in a tally sheet and encoded to Microsoft Excel using appropriate coding to facilitate statistical analyses. Descriptive statistics were employed where applicable. The statistical significance of the test results and profiles were evaluated using a Chi-square and Mann–Whitney U test. P values of less than 0.05 were considered significant. Odds ratio (OR) and confidence intervals (CI) were also computed.

### Ethical clearance

The study was conducted according to the principles of the Helsinki declaration developed by the World Medical Association. Written informed consent (S4 Fig) was obtained from the respondents or their representatives after a careful explanation of the study. Written permission from cat owners and rescue shelter workers (S5 Fig) was also sought before blood collection. Ethical clearance of this study in humans was reviewed and approved by the Institutional Review Board of the University of the Visayas, Cebu (UV-IRB2017-91). Sampling in animals was conducted in accordance with the Animal Welfare Act of the Philippines (RA 8485) and the Bureau of Animal Industry Administrative Order 40, and the protocol was reviewed and approved by the Gullas College of Medicine Institutional Animal Care and Use Committee (Protocol no. 2017-01-01).

## Results

Out of 924 participants, 244 (26.4%) were found to be seropositive (Table 1) from 21 different municipalities and cities in Cebu (Fig 1). Seropositivity was found to be greater among women (27%) than men (25.5%); however, sex was not found to be significantly associated with *T*. *gondii* seropositivity (*P* = 0.611). Students (28%) and respondents with non-health-related occupations (24.2%) were found to have higher seropositivity rates than health-related professionals (18.9%). Additionally, more seropositive participants had pet cats (35.2%) or were in frequent contact with these animals (45.4%). A higher proportion of seropositive individuals were suburban residents (35.1%) and consumed street foods (27.6%). Statistical analyses revealed no significant differences in seropositivity associated with sex or age. Furthermore, the respondents’ occupation was not significantly associated with seropositivity (*P* = 0.060). However, living in a suburban area (*P* = 0.002), cat ownership (*P* = 0.021), frequent contact with cats (*P* < 0.0001) and consumption of street foods (*P =* 0.044) were all found to be significantly associated with seropositivity to *T*. *gondii* in humans (Table 1). These factors were associated with an increased risk of being seropositive for *T*. *gondii* in humans with odds ratios of 1.66 (95% CI 1.20–2.31), 1.48 (95% CI 1.07–2.07), 2.43 (95% CI 1.73–3.42) and 1.58 (95% CI 1.01–2.48), respectively. Of the pregnant women tested in this study, 4 out of 18 (16.7%) were seropositive for *T*. *gondii* antibodies.

For cats, the seropositive detection rate was 42.3% (44 of 104), with seropositive cats detected in Liloan and Cebu City (Fig 2). Seropositivity was higher among stray cats (7/8; 87.5%) than household cats (21/47; 44.7%) (Table 2). However, the small sample size for stray cats in this study should be taken into consideration. Although the difference was not statistically significant (*P* = 0.400), more seropositive female cats (46.7%) were detected than male cats (38.6%). Greater seropositivity was observed in fertile cats (46%) than in neutered or spayed cats (36.6%). In this study, only one cat had poor body condition and this cat was negative for *T*. *gondii* antibodies. Body condition was found to have no significant association with seropositivity in cats (*P* = 0.623). The use of litter trays (*P* = 0.002) (Table 2) was found to be significantly associated with seropositivity in cats and increased the odds of cats becoming infected with *T*. *gondii* (OR 4.5, 95% CI: 1.73–11.71).

Serological testing revealed that 13.4% (69 of 514) of pigs were seropositive. Seropositive animals came from 10 municipalities/cities in Cebu (Fig 3). Most of the pigs that tested positive were female (14.1%) and were raised through non-intensive or backyard farming (14.3%) (Table 3). However, sex (*P* = 0.691) and the rearing system (*P* = 0.497) were not significantly associated with seropositivity in pigs.

## Discussion

The seroprevalence of *T*. *gondii* in humans in this study (26.4%) was slightly lower than the previously reported detection level (27.1%) in the Philippines using the same detection method [20]. Another report in the country showed a lower detection rate using ELISA on samples from Metro Manila (11.1%), but higher rates for Mindoro (61.2%) and Leyte (30.1%) [33]. The higher seropositivity rates among women in this study (27.0%) were similar to the findings of Salibay *et al*. [20] that reported 32% seropositivity. However, sex was not found to be significantly associated with seropositivity in previous or current studies in this country. The 16.7% detection rate among pregnant women in this study was comparable to that of previous studies in pregnant women in Singapore, which showed a prevalence of 17.2% [36], and in Vietnam with a seroprevalence of 11.2% [37], using immunofluorescent antibody test (IFAT) and ELISA, respectively. In Thailand, a slightly higher seroprevalence of 22% was reported in 2014 using ELISA [38]. Using the same method, 7.7% seropositivity was recently published in Taiwan [39], which is lower than that detected in the present study. However, the number of pregnant women tested was low, which is also a limitation of the current study. All of the above-mentioned reports did not find any significant association between seropositivity and risk factors, with the exception of the study by Andiappan *et al*. [38] that reported age, occupation (as laborers) and drinking water source as significant risk factors for *Toxoplasma* seropositivity in pregnant women in Thailand (*P* < 0.05).

Our study revealed no significant differences in the seropositivity rates relating to the age or sex of respondents, which was similar to previous findings in some areas of the Philippines [20,40]. Kawashima *et al*. [33], however, noted that infection rates in the Philippines increased with age. This implied that *T*. *gondii* may infect any sex at any age, but the frequency of seropositivity is higher among older individuals, most likely because they have been exposed to risk factors longer than the younger individuals. Previous studies in the Philippines reported significantly higher seroprevalence in rural and suburban areas than in urban areas [15,20], which was consistent with the findings of our study. This variance among locations may be attributed to the socioeconomic status of the participants, which may affect hygiene practices and the likelihood of ingestion of oocysts from water, crops, animal products and unpasteurized milk contaminated from oocysts shed by cats and contaminated soil [15,16]. A study in the Philippines also indicated higher seroprevalence among individuals with apparent association with cats [20]. Worldwide, the close association with, or ownership of cats have been identified as vital risk factors for human infection. Being the definitive host of *T*. *gondii*, cats have a primary role in the transmission of the parasite, and frequent contact with cats generally increases the risk of infection due to contact with oocysts in cat feces [18,19].

The reported serodetection of *Toxoplasma* in pregnant women in this study may have a potential detrimental effect on the fetus due to the risk of congenital infection. Congenital infection is a crucial part of the disease burden in human *Toxoplasma* infections [13]. This study is the first to serologically detect *T*. *gondii* infection in pregnant women in the Philippines. Unlike in other countries, screening is not routinely performed in this country. However, the widespread serodetection of *Toxoplasma* in this study implies the necessity for an increased sampling area and size to clarify the epidemiological status of this pathogen in the Philippines.

The serodetection rate observed in cats in this study is similar to that previously reported in the northern Philippines (46.67%) [30], but is lower than the reported seroprevalence of 72.3% in Vietnam [41]. A recent study in Japan, however, only documented *T*. *gondii* infection rate of 6.7% in cats [42]. Advincula *et al*. [30] found that male cats were more susceptible to infection; however, other reports, including this study, concluded that sex is not a risk factor for toxoplasmosis in cats [43–44]. The greater seropositivity observed in fertile cats in this study may be due to the behavioral habits of fertile cats to roam in search of a mate. Poor body condition was also reported to affect seropositivity in cats [25–26]. However, in this study, body condition showed no signification association with seropositivity in cats. Furthermore, the current study showed the increased likelihood of cats to become infected with *T*. *gondii* through the use of litter trays. According to the Center for Disease Control, exposure to cat litter trays may facilitate contact with infective *T*. *gondii* oocysts. Generally, location (urban, suburban and rural areas), diet and domestication influence seropositivity in animals [27–28]. Seropositivity for infection was significantly higher in suburban than in urban areas for cats in Japan [42] and was also higher among strays than domesticated cats and dogs in Korea and in other parts of the Philippines [29,45–46], which is consistent with the results of this study.

In 1982, the first and only report of toxoplasmosis in Cebu, Philippines, documented a 29.50% (59 of 200) seropositivity rate among slaughter pigs [35]. Our study investigated the current infection status in swine in Cebu. The seroprevalence rate in pigs was found to be lower than the initial report by Manuel [35], as well as the prevalence (27.2%) reported in Vietnam [47]. However, it was similar to a previous report in Metro Manila (13.6%) [48] and reports from other neighboring countries, such as Taiwan (10.1%) [49] and China (10.4%) [50]. Lower seroprevalence was reported in Indonesia and Japan at 2.3% and 5.2%, respectively [51–52]. The differences in seroprevalence reported by these studies may be attributed to the various serodiagnostic techniques and cut-off values employed [23]. Although the rearing system was not significantly associated with seropositivity in pigs in this study (*P* = 0.500), the method by which pigs are farmed affects seroprevalence by potentially predisposing the animals to infection [23,24]. The substantially higher serodetection rate among pigs farmed by extensive (non-intensive/backyard) production methods compared with intensively farmed pigs is due to the access of these animals to the outdoors, which increases contact with infected rodents and dead animals and oocyst-contaminated feed, water and surroundings, as reported in the USA and Japan [2,24,53]. Access of cats to farm animals such as pigs has also been identified as a critical risk factor for the transmission of *T*. *gondii* infection [54]. The serological detection of *T*. *gondii* in pigs in Cebu three decades after its first detection revealed the constant presence of *T*. *gondii* in the area. In general, seropositivity gauges the presence of *T*. *gondii* in tissues [2,55]. The findings of our study suggest the endemicity of *T*. *gondii* infection in pigs reared in Cebu. This presents a public health hazard, as *T*. *gondii* cysts in pork may be infectious if the meat is undercooked.

The prevalence rates obtained in this study were dependent on the detection method used. The LAT detects total antibody and does not distinguish between recent and past exposures. The kit used in this study had been used in several serological surveys with various animal species including humans because of its ease of use, low cost, and high sensitivity, and specificity (20,52). Because the performance of assays can differ, relative seroprevalence rates may also vary depending on the test used. Thus, statistical correlations on risk factors may also give varying results depending on the assay used in a study.

While several other countries are aware of the disease and have already implemented routine screening for toxoplasmosis, most communities in the Philippines remain unaware of this disease. It is estimated that only 11% of pregnant women in the Philippines are aware of the disease [38]. The positive serodetection rates among pregnant women in this study suggest the possible presence of infection in other parts of the Philippines. While serological evidence of exposure of pigs, humans and cats to *T*. *gondii* was presented, the present study could not account for the status of the disease across the entire country because of sampling limitations. Future studies with an increased sampling area and size could help clarify the epidemiological status of *T*. *gondii* in this country and determine whether screening among the general population is essential. As our study detected seropositive cats in areas with confirmed cases of seropositive humans, there is a need to conduct further studies in stray cats that are commonly found in areas selling street foods, with transmission possibly resulting from the consumption of infected rats or meat. Moreover, seropositivity was also detected among pigs in the country, which places the predominantly meat-eating Filipino people at higher risk of consumption of potentially undercooked infected meat. Food preparation, hygienic and sanitary practices may also lead to the contamination of food from uncooked contaminated meat and other contaminated food.

## Conclusions

The present study reports the first serological detection of *T*. *gondii* in humans and cats in Cebu, Philippines, and the most recent serological status update in pigs since 1982 in the area. To the best of our knowledge, this is also the first report documenting serodetection of *T*. *gondii* in pregnant women in the country. The confirmed seropositivity of *T*. *gondii* in Cebu, Philippines, in the present study implies the endemicity of this disease in the area and highlights the need for increased public awareness in the country. Routine testing may be recommended for vulnerable populations, including pregnant women, immunocompromised individuals and those exposed to cats. An educational campaign about toxoplasmosis is needed to increase awareness among the public.

## Supporting information

S1 Fig Questionnaire used during sample collection with respondents. The questionnaire was used to obtain the profile of the respondents which was used during the analysis.
(PDF)

S2 Fig Questionnaire used during sample collection in cats. The questionnaire was used to obtain the profile of the cats which was used during the analysis.
(PDF)

S3 Fig Questionnaire used during sample collection in slaughter pigs. The questionnaire was used to obtain the profile of the pigs which was used during the analysis.
(PDF)

S4 Fig Written consent form given to the respondents. A signed informed consent was obtained from the respondents after careful explanation of the study.
(PDF)

S5 Fig Written permission sent to rescue shelter owners. Letter was sent to cat owners and rescue shelter owners to seek approval prior to blood collection.
(PDF)

S1 Data Raw data used for analysis in humans. Excel file of raw data obtained for humans.
(XLSX)

S2 Data Raw data used for analysis in cats. Excel file of raw data obtained for cats.
(XLSX)

S3 Data Raw data used for analysis in slaughter pigs. Excel file of raw data obtained for slaughter pigs.
(XLSX)
