# Supplementary material for: Endemicity of Toxoplasma infection and its associated risk factors in Cebu, Philippines
Source: PLoS One. 2019 Jun 12;14(6):e0217989. doi: 10.1371/journal.pone.0217989 (PMC6561560; doi:10.1371/journal.pone.0217989)
Supplement: S1 Fig — The questionnaire was used to obtain the profile of the respondents which was used during the analysis. (PDF) [file pone.0217989.s001.pdf]

ID No. ____________

Seroprevalence of *Toxoplasma gondii* in humans in Cebu

Main Researchers/Project Leaders: **Rochelle Haidee D. Ybañez[1] and Adrian P. Ybañez[1,2]**
[1]Biology and Environmental Studies Program, Sciences Cluster, University of the Philippines Cebu, Gorordo Avenue, Lahug, Cebu City
[2]Gullas College of Medicine, University of the Visayas, Gov. M. Cuenco Ave, Cebu City

Profile (Please provide the necessary information or mark √ to applicable answers)

Name: ____

Address: ____

Age: _____ years Sex: □ Male □ Female Civil Status: □ single □ married □ widowed

Highest Educational Attainment: □ Elementary Level □ Elementary Graduate
□ High School level □ High School Graduate
□ College Level □ College Graduate
□ Others (please specify) _________________________

Profession/Work: ____

Present medical conditions: □ None □ Others (Please indicate) _____________________________________

Previous medical conditions/procedures: □ None □ Others (Please indicate) _________________________

Do you own a cat? □ No □ Yes (please indicate number) ____________

If yes, how long have you been living with cats? ______ years

Do you have constant contact with cats? □ No □ Yes (please estimate or describe frequency)

____

Do you own other animals aside from cats? □ No □ Yes (please indicate) ____________________________

Do you eat street foods? □ No □ Yes (indicate where) ____

Are you currently pregnant? □ No □ Yes
