# Supplementary material for: Endemicity of Toxoplasma infection and its associated risk factors in Cebu, Philippines
Source: PLoS One. 2019 Jun 12;14(6):e0217989. doi: 10.1371/journal.pone.0217989 (PMC6561560; doi:10.1371/journal.pone.0217989)
Supplement: S2 Fig — The questionnaire was used to obtain the profile of the cats which was used during the analysis. (PDF) [file pone.0217989.s002.pdf]

Sample No. ___________

Serological survey of toxoplasmosis in cats in Cebu, Philippines

Type of cat: □ Household/Domesticated □ Stray/In the shelter
Address: ____________________________________
Age: _____ years
Sex: □ Male (Neutered /Not) □ Female (Spayed /Not)
Body Condition: □ Good □ Bad
Type of diet: □ Commercial food □ Table food □ Raw meat □ Mixed ______________
Use of litter tray (for feces): □ Yes □ No
Access to outdoors/hunting: □ Yes □ No
Presence of dogs or other cats in the household/shelter: □ Yes □ No

---

Sample No. ___________

Serological survey of toxoplasmosis in cats in Cebu, Philippines

Type of cat: □ Household/Domesticated □ Stray/In the shelter
Address: ____________________________________
Age: _____ years
Sex: □ Male (Neutered /Not) □ Female (Spayed /Not)
Body Condition: □ Good □ Bad
Type of diet: □ commercial food □ table food □ raw meat □ Mixed ______________
Use of litter tray (for feces): □ Yes □ No
Access to outdoors/hunting: □ Yes □ No
Presence of dogs or other cats in the household/shelter: □ Yes □ No

---

Sample No. ___________

Serological survey of toxoplasmosis in cats in Cebu, Philippines

Type of cat: □ Household/Domesticated □ Stray/In the shelter
Address: ____________________________________
Age: _____ years
Sex: □ Male (Neutered /Not) □ Female (Spayed /Not)
Body Condition: □ Good □ Bad
Type of diet: □ commercial food □ table food □ raw meat □ Mixed ______________
Use of litter tray (for feces): □ Yes □ No
Access to outdoors/hunting: □ Yes □ No
Presence of dogs or other cats in the household/shelter: □ Yes □ No
