# Supplementary material for: Endemicity of Toxoplasma infection and its associated risk factors in Cebu, Philippines
Source: PLoS One. 2019 Jun 12;14(6):e0217989. doi: 10.1371/journal.pone.0217989 (PMC6561560; doi:10.1371/journal.pone.0217989)
Supplement: S3 Fig — The questionnaire was used to obtain the profile of the pigs which was used during the analysis. (PDF) [file pone.0217989.s003.pdf]

Sample No. ___________

Serological survey of toxoplasmosis in slaughter pigs in Cebu, Philippines

Location of Source of Pigs: _______________________________
Rearing system: □ Intensive (Closed environment) If intensive: □ Conventional □ Organic
□ Non-intensive (Open/Backyard)
Age: _____ months (estimate if exact age is unknown)
Sex: □ Male □ Female

Sample No. ___________

Serological survey of toxoplasmosis in slaughter pigs in Cebu, Philippines

Location of Source of Pigs: _______________________________
Rearing system: □ Intensive (Closed environment) If intensive: □ Conventional □ Organic
□ Non-intensive (Open/Backyard)
Age: _____ months (estimate if exact age is unknown)
Sex: □ Male □ Female

Sample No. ___________

Serological survey of toxoplasmosis in slaughter pigs in Cebu, Philippines

Location of Source of Pigs: _______________________________
Rearing system: □ Intensive (Closed environment) If intensive: □ Conventional □ Organic
□ Non-intensive (Open/Backyard)
Age: _____ months (estimate if exact age is unknown)
Sex: □ Male □ Female

Sample No. ___________

Serological survey of toxoplasmosis in slaughter pigs in Cebu, Philippines

Location of Source of Pigs: _______________________________
Rearing system: □ Intensive (Closed environment) If intensive: □ Conventional □ Organic
□ Non-intensive (Open/Backyard)
Age: _____ months (estimate if exact age is unknown)
Sex: □ Male □ Female
