# Supplementary material for: Endemicity of Toxoplasma infection and its associated risk factors in Cebu, Philippines
Source: PLoS One. 2019 Jun 12;14(6):e0217989. doi: 10.1371/journal.pone.0217989 (PMC6561560; doi:10.1371/journal.pone.0217989)
Supplement: S4 Fig — A signed informed consent was obtained from the respondents after careful explanation of the study. (PDF) [file pone.0217989.s004.pdf]

Prevalence of *Toxoplasma gondii* in humans in Cebu

Project Leaders/ Main Researchers: Rochelle Haidee D. Ybañez[1] and Adrian P. Ybañez[1,2]
[1]Biology and Environmental Studies Program, Sciences Cluster, University of the Philippines Cebu, Gorordo Avenue, Lahug, Cebu City
[2]Gullas College of Medicine, University of the Visayas, Gov. M. Cuenco Ave, Cebu City

Informed Consent Form for Adult Participants/ Volunteers

This Informed Consent Form has two parts:

- **Information Sheet (to share information about the study with you)**
- **Certificate of Consent (for signatures if you choose to participate)**

You will be given a copy of the full Informed Consent Form upon request

Part I: Information Sheet

Introduction

We are researchers who are studying the disease Toxoplasmosis. We are going to give you information and invite you to be part of this research. You do not have to decide today whether or not you will participate in the research. Before you decide, you can talk to anyone you feel comfortable with about the research.

This consent form may contain words that you do not understand. Please ask us to stop as we go through the information and we will take time to explain. If you have questions later, you can ask them from us.

Purpose of the research

Toxoplasmosis is a zoonotic disease caused by *Toxoplasma gondii*, and transmitted through the feces of the cat. It can cause abortions in pregnant women, neurological sign, and/or complications for those that are having a weakened immune system (e.g. cancer. HIV/AIDS). We want to know the prevalence of this disease in the area, and assess risk factors that maybe associated with its occurrence to recommend possible preventive measures.

Type of Research Intervention

The research will involve your participation in answering the questionnaire that will take less then 30 minutes, and in obtaining blood sample for testing.

Participant Selection

You are being invited to participate in this research because we believe that your participation can contribute much to our understanding and knowledge on the current status of toxoplasmosis in the area.

Voluntary Participation

Your participation in this research is entirely voluntary. It is your choice whether to participate or not. You may change your mind later and stop participating even if you agreed earlier.

Procedures

We are requesting you to help us learn about Toxoplasmosis in the area. We are inviting you to take part in this research project. I f you accept, you will be asked to answer the questionnaire, and have a blood sample taken from you, which will be serologically tested for toxoplasmosis. As for the questionnaire, it will be provided by the researchers, OR you may answer the questionnaire yourself, OR it can be read to you and you can say out loud the answer you want me to write down.

If you do not wish to answer any of the questions, you may skip them and move on to the next question. The information recorded is confidential, your name is not being included on the forms, only a

number will identify you, and no one else except the researchers will have access to your survey.

Duration

Questionnaire/interview will be short (around 30 minutes), while blood sampling will take around 3 minutes. The results of the serological testing will be provided to you in not more than 2 weeks after the blood sample is collected.

Risks

There is no major potential risks from the research, other than the possible information which you may deem personal. If you feel uncomfortable, you do not have to answer any question or take part in the discussion/interview/survey if you don't wish to do so, and that is also fine. You do not have to give us any reason for not responding to any question, or for refusing to take part in the interview.

Benefits

Other than knowing your status if you are positive with toxoplasmosis or not, there will be no direct benefit to you, but your participation is likely to help us find out more about the status of toxoplasmosis in the area and on how to prevent it in the community.

Reimbursements

You will not be provided any incentive to take part in the research. However, we will share with you your test results. The testing approximately costs PHP 1,500 to 2,500.

Confidentiality

The research may draw attention and if you participate, you may be asked questions by other people in the area. We will not be sharing information about you to anyone outside of the research team. The information that we collect from this research project will be kept private. Any information about you will have a number on it instead of your name. Only the researchers will know what your number is and we will lock that information up with a lock and key. It will not be shared with or given to anyone except the research sponsors.

Sharing the Results

Nothing that you tell us today will be shared with anybody outside the research team, and nothing will be attributed to you by name. The knowledge that we get from this research will be shared with you and your community before it is made widely available to the public. Each participant will receive a summary of the results. We will publish the results so that other interested people may learn from the research.

Right to Refuse or Withdraw

You do not have to take part in this research if you do not wish to do so, and choosing to participate will not affect your job or job-related evaluations in any way. You may stop participating in the interview/questionnaire at any time that you wish. We will give you an opportunity at the end of the interview/discussion to review your remarks, and you can ask to modify or remove portions of those, if you do not agree with our notes or if we did not understand you correctly.

Who to Contact

If you have any questions, you can ask them now or later. If you wish to ask questions later, you may contact any of the following: Adrian P. Ybanez, dr.adrianpybanez@gmail.com / 09228762439; Rochelle Haidee D. Ybanez, rdybanez1@up.edu.ph (09258237423).

This proposal has been reviewed and approved by UV-IRB, which is a committee whose task it is to make sure that research participants are protected from harm. If you wish to find about more about the IRB, contact Marites G. Arcilla, RN, MAN, DPE, DMc of the University of the Visayas. It has also been reviewed by CHED.

Part II: Certificate of Consent

I have been invited to participate in research about Toxoplasmosis. I have read the foregoing information about the research, or it has been read to me. I have had the opportunity to ask questions about it and any

questions I have been asked have been answered to my satisfaction. I consent voluntarily to be a participant in this study

Print Name of Participant (optional) ____________________
Signature of Participant ____________________
Date ____________________________
Day/month/year

If illiterate [1]

I have witnessed the accurate reading of the consent form to the potential participant, and the individual has had the opportunity to ask questions. I confirm that the individual has given consent freely.

Print name of witness ____________________ Thumb print of participant

Signature of witness ____________________

Date _______________________
Day/month/year

Statement by the researcher/person taking consent

I have accurately read out the information sheet to the potential participant, and to the best of my ability made sure that the participant understands that the following will be done:
1. answering of the questionnaire
2. blood collection for testing with Toxoplasmosis3.
I confirm that the participant was given an opportunity to ask questions about the study, and all the questions asked by the participant have been answered correctly and to the best of my ability. I confirm that the individual has not been coerced into giving consent, and the consent has been given freely and voluntarily.

A copy of this ICF can be provided to the participant upon request.

Rochelle Haidee D. Ybañez/ Adrian P. Ybañez
Researchers

Date ____________________________
Day/month/year

[1] A literate witness must sign (if possible, this person should be selected by the participant and should have no connection to the research team). Participants who are illiterate should include their thumb print as well.
