# Supplementary material for: Endemicity of Toxoplasma infection and its associated risk factors in Cebu, Philippines
Source: PLoS One. 2019 Jun 12;14(6):e0217989. doi: 10.1371/journal.pone.0217989 (PMC6561560; doi:10.1371/journal.pone.0217989)
Supplement: S5 Fig — Letter was sent to cat owners and rescue shelter owners to seek approval prior to blood collection. (PDF) [file pone.0217989.s005.pdf]

February 6, 2018

MR. DAN VETTER
Vice President
Mayari Animal Rescue Organization, Inc.

Dear Mr. Vetter,

Good day! I am a friend of Ms. Deena Kelly. I am currently pursuing my doctorate degree in Obihiro University of Agriculture and Veterinary Medicine in Japan. My research is on the development of serodiagnostic antigens of *Toxoplasma gondii* for its epidemiological study in the Philippines. Just to give you a brief background, *Toxoplasma gondii* is a microscopic protozoan parasite that is ubiquitous in nature. It causes the disease called Toxoplasmosis, which is considered one of the most common parasitic diseases around the world.

It is usually asymptomatic to healthy individuals or they may only develop flu-like symptoms because the immune system keeps the parasite from causing any illness. However, the disease is more problematic for pregnant women (causes abortion and/or abnormality in the babies such as hydrocephaly, brain or eye damage) and people who have weakened immune systems (causes brain disease, brain lesions, pneumonitis or retinochoroiditis). The parasites remain in the body as tissue cysts (bradyzoites) and reactivate, if the person becomes immunosuppressed by other diseases or by immunosuppressive drugs. Cats are the primary host of the parasite and humans and other warm blooded animals can be intermediate hosts.

Humans get toxoplasmosis through:

- consumption of undercooked, infected meat (especially pork, lamb, and chevon)
- consumption of fruits and vegetables or ingesting water, soil (i.e., putting dirty fingers in your mouth) or anything else that has been contaminated with cat feces
- mother-to-child transmission
    - A pregnant woman might not have any symptoms, but the unborn child might suffer and develop the disease.
- blood transfusion or organ transplantation (very rare)

In other countries, routine testing has already been conducted especially among pregnant women. However, not a lot of people know about toxoplasmosis in the Philippines. We have already conducted preliminary serological screening among veterinary personnel and pet owners in Cebu and other parts of the country. And there were individuals that tested positive of antibodies against the parasite. Those pet owners that tested positive had cats as pets.

In this regard, I would like to request for your kind approval to allow us to extract blood samples from the cats in the shelter so we could test them for toxoplasmosis. We have a team of veterinarians who can help facilitate the blood extraction for our cats and we can tap a phlebotomist to do the blood extraction for you and your personnel and volunteers should you decide to have your blood tested as well.

Your kind support and participation will really be of great help for the fulfillment of this research endeavor.

Thank you very much.

Respectfully yours,

ROCHELLE HAIDEE D. YBAÑEZ
